# GAAP: A Genome Assembly + Annotation Pipeline

**DOI:** 10.1155/2019/4767354

**Published:** 2019-06-26

**Authors:** Jinhwa Kong, Sun Huh, Jung-Im Won, Jeehee Yoon, Baeksop Kim, Kiyong Kim

**Affiliations:** ^1^Korea National Institute of Health, Cheongju 28159, Republic of Korea; ^2^Department of Parasitology and Institute of Medical Education, Hallym University, Chuncheon 24252, Republic of Korea; ^3^Smart Computing Lab., Hallym University, Chuncheon 24252, Republic of Korea; ^4^School of Software, Hallym University, Chuncheon 24252, Republic of Korea; ^5^Department of Electronic Engineering, Kyonggi University, Suwon 16227, Republic of Korea

## Abstract

Genomic analysis begins with* de novo* assembly of short-read fragments in order to reconstruct full-length base sequences without exploiting a reference genome sequence. Then, in the annotation step, gene locations are identified within the base sequences, and the structures and functions of these genes are determined. Recently, a wide range of powerful tools have been developed and published for whole-genome analysis, enabling even individual researchers in small laboratories to perform whole-genome analyses on their objects of interest. However, these analytical tools are generally complex and use diverse algorithms, parameter setting methods, and input formats; thus, it remains difficult for individual researchers to select, utilize, and combine these tools to obtain their final results. To resolve these issues, we have developed a genome analysis pipeline (GAAP) for semiautomated, iterative, and high-throughput analysis of whole-genome data. This pipeline is designed to perform read correction,* de novo* genome (transcriptome) assembly, gene prediction, and functional annotation using a range of proven tools and databases. We aim to assist non-IT researchers by describing each stage of analysis in detail and discussing current approaches. We also provide practical advice on how to access and use the bioinformatics tools and databases and how to implement the provided suggestions. Whole-genome analysis of* Toxocara canis* is used as case study to show intermediate results at each stage, demonstrating the practicality of the proposed method.

## 1. Introduction

Recent technological advances in next-generation sequencing (NGS) have dramatically reduced the cost of producing short reads of genomes of new species. Recent developments in the various bioinformatics tools used for sequencing data have enabled small-scale laboratories to perform analyses such as preprocessing,* de novo* assembly, gene prediction, and functional study.

While sequencing procedures have been made straightforward, genomic analysis has grown more difficult and challenging. Several factors are responsible for this. First, NGS techniques produce short reads; when these reads are used for* de novo* assembly, the accuracy of the assembled base sequences typically declines to the level of a draft genome. Second, for newly sequenced genomes, there are no gene models to serve as a reference; therefore, it is difficult to ensure the accuracy of annotation. Third, annotation of the same genome is performed by various research groups using different analysis tools and annotation methods. This necessitates combining all the results to obtain a final consensus annotation. Fourth, genomic analysis is often conducted on a small-scale by researchers who have little expertise in bioinformatics and computational biology. Although small-scale genomic analysis is currently within the reach of nonexperts, it remains a challenging task [1].

In this study, we describe the implementation of GAAP, a genome analysis pipeline that enables small laboratories to successfully perform genome assembly and annotation using the relatively short-read data generated by cost-effective NGS technologies. The proposed pipeline consists of a sequencing stage, an assembly stage, and an annotation stage performed using a range of proven tools and databases.

In a previous study [2], we determined the genomic sequence of* Toxocara canis* (*T. canis*) using next-generation sequencing,* de novo* assembly, and annotation. Here, we use* T. canis* to illustrate the intermediate results of* T. canis* genome analysis derived using the step-by-step protocol for this pipeline. We provide guidance for each stage of genomic analysis, describe the available software tools and databases, and outline best-practice approaches. With our system, researchers without background in IT can access public software and biological databases to easily conduct a complete genomic analysis including structures and functions of genes of interest.

## 2. Methods and Results

**Figure 1:**
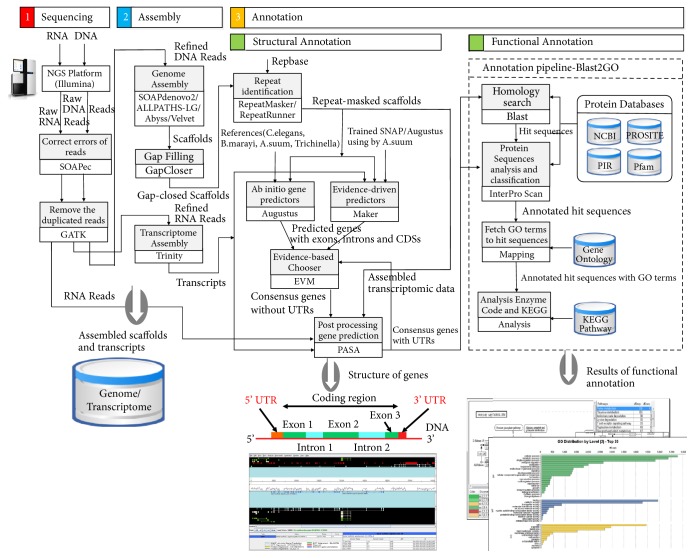
Overview of the genome analysis process of the GAAP pipeline system. The overall workflow of the system is shown, and all software tools and annotation databases are summarized.

Figure 1 shows a workflow of the GAAP pipeline described in this study. The figure shows the flow of input/output data, all software tools, and annotation databases used in this system. The process of genome analysis is divided into stages of sequencing, assembly, and annotation. In the sequencing stage, an NGS platform is used to produce DNA/RNA reads, and error-correction tools are used to refine the reads. In the assembly stage, the sequencing reads are assembled to obtain scaffolds, after which gap filling is performed to improve the accuracy of the scaffolds. The annotation stage is further divided into structural annotation and functional annotation. In structural annotation, repeated sequences in the assembled scaffolds are masked. Then, a gene prediction tool is used to locate genes within the scaffolds, and the structures of introns, exons, and untranslated regions (UTRs) constituting these genes are determined. Finally, in functional annotation, homology search and ontology mapping are performed using structure-annotated sequences in order to determine the functions of the genes.

### 2.1. Sequencing and Error Correction

The accuracy of sequencing data directly affects the accuracy of assembly results, analysis of gene structure and function, and protein analysis. NGS platforms include Roche/454 (https://sequencing.roche.com), Illumina (http://www.illumina.com), SOLiD [3], Pacific Biosciences (https://www.pacb.com), and Ion Torrent (https://www.lifetechnologies.com/iontorrent). Sequence reads generated by these platforms possess different characteristics such as read length, type, and rate of errors. SOLiD and Illumina generate relatively short reads of 100-300 bp with low rates of error. Conversely, Pacific Biosciences generates long reads of over 15 Kbp, but has a proportionally higher rate of error (Supplementary Table S1). For Illumina, most errors are substitution errors; for Roche/454, Ion Torrent, and Pacific Biosciences, most are indel errors. De Bruijn assembly tools are known to be effective for correcting substitution errors, while overlap-layout-consensus (OLC) assembly tools are more effective for correcting indel errors. Therefore, it is important to select the appropriate assembly tool to fit the properties of sequencing data [4].

Typically, longer reads are more advantageous than shorter reads because longer reads simplify the assembly process and provide more accurate genome analysis. However, the long reads generated by platforms, such as Pacific Biosciences, are very costly; this highlights the need to generate a large number of paired-end reads of approximately 100 bp in length for assembly and annotation. In this study, we assumed that the assembly and annotation processes would use large-scale paired-end reads of approximately 100-bp using Illumina.

When short reads are used, it is essential to generate various read datasets in order to obtain accurate results for genome analysis. If the read length is too short, it may be impossible to determine whether the reads are actually generated from repeat sequence regions; this increases the rate of false positive overlap and can render genome analysis based on short reads very difficult, incomplete, or impossible [5–7]. For repeat sequence regions, which are poorly analyzed using only short reads, it is important to ensure sufficient coverage with reads of different insert sizes. For example, reads with a very short insert size generate overlapping paired-read sequences, which may result in reads longer than the original read length. Mate-pair reads refer to paired-end reads with a long insert size. While paired-end reads have an insert size of 300–500 bp between the pairs, mate-pair reads have an insert size of 2–10 Kbp. Mate-pair reads are advantageous because they can improve the accuracy of genome analysis; the information about the direction and distance between two paired sequences can be used to detect repeat sequences, errors, and structural variations during assembly, or to generate longer scaffold sequences by joining contig sequences.

After sequencing has been completed, read sequence data and information about quality are conveyed to the user in a text file using FASTQ format. However, raw-read data typically include numerous errors from the sequencing process; therefore, these errors need to be removed by the correction process. Correction tools can be broadly divided into k-mer-based tools, suffix tree/array-based tools, and multiple-sequence alignment (MSA)-based tools. K-mer based tools extract all the substrings of length k (k-mers) from the reads and correct the errors using k-mer frequencies. These tools are useful for substitution errors [8–10]. Suffix tree/array-based tools, such as SHREC [11] and HiTEC [12], are useful for indel errors; here, suffixes are extracted from all reads and their reverse complements, stored in a tree or array structure, and errors are corrected based on thorough statistical analysis of suffixes. MSA-based tools, such as Coral [13] and ECHO [14], retrieve all reads that share at least one k-mer and perform a multiple-sequence alignment of these reads, which probably come from same genomic locus. Using this alignment, a consensus sequence is generated and used as the new reference to be aligned with the remaining reads. Once the MSA is completed, the reads are corrected for the consensus sequence of the alignment.

GAAP uses SOAPec (https://sourceforge.net/projects/soapdenovo2/files/ErrorCorrection), which is a k-mer-based error-correction tool.  Table 1 shows how to use the SOAPec commands. The error-correction process consists of two stages: first, k-mer frequency spectrum is generated by running KmerFreq_AR on readlist.txt, which contains a list of read files used as input data. Here, the options -k, -t, and -p correspond to k-mer size, thread count, and prefix of the output file, respectively. The output of KmerFreq_AR consists of: prefix.freq.cz, which is the k-mer frequency file; prefix.freq.cz.len, which stores data on the length of each block in the k-mer frequency file; and prefix.freq.stat, which contains statistical information about k-mer frequency. Next, errors are corrected by running Corrector_AR. Here, the options -k, -l, -r, and -t correspond to k-mer size, cutoff size for removing low- frequency k-mers, minimum length of reads, and thread count, respectively. The input files consist of readlist.txt, prefix.freq.cz, and prefix.freq.cz.len from the output of KmerFreq_AR. The output of Corrector_AR for each read file consists of: *∗*.cor.pair_*∗*.fq, which contains paired-end reads and stores newly refined reads; *∗*.cor.stat, which stores statistical information; and readlist.txt.QC.xls, which stores information on the read quality control.

In order to improve the accuracy of assembly and annotation, GAAP uses Picard (https://broadinstitute.github.io/picard) to remove duplicate reads. Tables 2 and 3 show examples of using the commands. First, as shown in Table 2, the FastqToSam command is used to convert the error-corrected FASTQ file, prefix.fq, into a SAM/BAM file. Here, for the options F1 and F2, paired-end reads in two read files are indicated by F1 and F2, while single-end reads in a single file are indicated by F1. Option O is used to indicate the output file, and SM is the sample name to be inserted into the header of the SAM/BAM file. Next, the Markduplicates command is used on the converted SAM/BAM read file, as shown in Table 3, in order to remove duplicate reads. Here, option I is the SAM/BAM read file generated from the procedure in Table 2. Option O is the output read file generated after removing duplicate reads. Option M is the duplication metrics file, which stores information summarizing the process of duplicate removal, including the number of mapped reads and number of duplicate reads. REMOVE_DUPLICATES is used to choose whether to output the duplicated reads to the file designated in option O; if the value is true, the duplicates are not included in the output

### 2.2. *De Novo* Assembly

#### 2.2.1. Genome Assembly

*De novo* assembly refers to the process of using short overlapping reads to obtain a genome sequence for a species without a reference sequence. The assembly process yields two different sequences: contigs and scaffolds.

The* de novo* assembly methods using NGS techniques are divided into the greedy graph method, overlap-layout-consensus method, and the De Bruijn graph method [4]. These methods are used to search for the longest overlapping regions between different reads; these regions are gradually joined together to form contigs. Assembly tools that use the greedy graph method include SSAKE [15], SHARCGS [16], and VCAKE [17]. They follow a simple, but effective, strategy in which the assembler greedily joins the reads that are most similar to each other. However, because only local information is considered at each step, the assembler can be easily confused by complex repeats, leading to misassembled contigs.

In the overlap-layout-consensus method, the relationships between the reads can be represented as an overlap graph; here, the nodes represent each of the reads, and an edge connects two nodes if the corresponding reads overlap. The algorithm determines the best path through the graph that contains all the nodes; this is called a Hamilton path. Assembly tools based on overlap-layout-consensus methods include Newbler [18], Celera assembler [19], and Edena [20].

The De Bruijn graph shows a compact representation based on k-mers, not reads; thus, high redundancy is handled by the graph without affecting the number of nodes. Each repeat is presented only once in the graph, with explicit links to the different start and end points. One advantage of this approach is that repeats are easily recognizable, while in an overlap graph repeats are more difficult to identify. This approach is adopted by several leading assemblers, including SOAPdenovo2 (https://github.com/aquaskyline/SOAPdenovo2) [10, 21], ALLPATHS (http://software.broadinstitute.org/allpaths-lg/blog/?page_id=12) [22, 23], Abyss (http://www.bcgsc.ca/platform/bioinfo/software/abyss) [24, 25], and Velvet (http://www.ebi.ac.uk/~zerbino/velvet) [26]. With these publicly available assembly tools, large-scale, high-coverage data generally have to be loaded into memory. Drawbacks to this include requirement for a high-performance computer installed with a large memory, and having very large temporal and spatial overheads. The accuracy of assembly results is affected by read insert size and k-mer size settings; therefore, optimal parameter settings are crucial. GAAP employs four assembly tools, SOAPdenovo2 [21], ALLPATHS-LG [23], Abyss 2.0 [25], and Velvet [26], which are widely used. User can adopt one of these tools by considering the assembly quality, memory requirement, and execution time [27–30].

*(1) SOAPdenovo2*. SOAPdenovo can perform assembly even for large mammalian genomes while using relatively small memory. In addition, its successor, SOAPdenovo2 (referred to here as SOAPdenovo), includes improvements and new features, including the new contig and scaffold construction improvements [21].

Before running the SOAPdenovo command, it is first necessary to generate a configuration file. The data are usually organized as multiple read files generated from multiple libraries.  Table 4 shows an example of a configuration file, which has multiple library sections. In this file, q1 and q2 indicate the read files with FASTQ formats prepared for assembly. Max_rd_len represents the maximum read length, while avg_ins represents the mean insert size of the reads. Reverse_seq represents how the read sequences need to be complementary reversed. The option value is set to 0 (forward-reverse) for paired-end reads with an insert size of 500 bp or less, and is set to 1 (reverse-forward) for paired-end reads with an insert size of at least 2 kbp. Rank value represents the order in which read files are used for scaffold assembly. For example, read files with the same rank value are used at the same time during assembly. Asm_flags indicates which parts of the assembly process use the read file. Asm_flags accepts the value of 1 (for contig assembly only), 2 (for scaffold assembly only), 3 (for both contig and scaffold assembly), or 4 (for gap closure only). In the example in Table 4, using reads with an insert size of 170, asm_flags is set to 3. This means that the corresponding read data set will be used to generate both contigs and scaffolds. After choosing the settings, Table 5 shows how to run the SOAPdenovo command using the configuration file. Here, -K is used to set the k-mer length, and -o is used to set the corresponding prefix of the output file name. The output of SOAPdenovo consists of outprefix.contig files (which store the contigs) and outprefix.scafSeq files, which store the scaffolds generated by* de novo* assembly.

*(2) ALLPATHS-LG*. ALLPATHS‐LG is able to perform* de novo* assembly of large mammalian genomes [23]. It has been designed to use reads generated on the Illumina platform, and optimized for reads of approximately 100-bp. However, ALLPATHS‐LG (referred to here as ALLPATHS) requires a minimum of 2 paired‐end libraries – one fragment library with overlapping paired-end reads and one jumping library with long insert size.

Before running the ALLPATHS command, it is first necessary to gather the read data in the appropriate formats, and then add metadata to describe them. The perl script PrepareAllPathsInputs.pl as shown in Table 6 can be used to automatically convert a set of BAM, fasta, fastq, or fastb files to ALLPATHS input files. Here, DATA_DIR is the location of the ALLPATHS DATA directory where the converted reads will be placed, and PLOIDY option is used to generate the ploidy file. In addition, the user must provide two metadata files (in_groups.csv and in_libs.csv), which describe the locations and library information of the various files to be converted, respectively. Each line in in_groups.csv corresponds to a BAM or fastq file to be imported, and ties each file to a library. Each line in in_libs.csv describes the detailed information of each library. The options IN_GROUPS_CSV and IN_LIBS_CSV determine where these data are found.

Table 7 shows how to run the RunAllPathsLG command using the imported data. As ALLPATHS actually uses a number of different k-mer sizes internally, the user cannot adjust the k-mer size from the default value of 96, unlike in many other assemblers. Here, the command-line argument PRE is used to specify the location of the root directory in which ALLPATHS pipeline directory will be created. REFERENCE_NAME is used to specify the REFERENCE (organism) directory name, which should be set to the same value of organism_name as given in in_libs.csv. DATA_SUBDIR is used to specify the location of the DATA directory, which contains all the converted read data. The RUN directory contains all the intermediate files, and the SUBDIR directory is where the final assembly is generated, along with some evaluation files. The results of the assembly are given in the following two files, final.assembly.fasta and final.assembly.efasta. Both these files contain the final flattened and scaffolded assembly. The efasta, “enhanced” fasta, file is a new format used by ALLPATHS and is based on the standard fasta file format.

*(3) Abyss*. Abyss is a multistage* de novo* assembly tool consisting of unitig (De Bruijn graph), contig, and scaffold stages. The recently published program Abyss 2.0 includes a Bloom filter-based implementation of the unitig assembly stage, which reduces the overall memory requirements [25].  Table 8 shows how to run the Abyss command using the paired-end reads. The input data are usually organized as multiple read files generated from multiple libraries. The names of the paired-end libraries and mate-pair libraries are specified using the parameters lib and mp, respectively. A pair of reads must be named with the suffixes 1 and 2 to identify the first and second read.  Table 8 shows an example of assembling a dataset with two paired-end libraries (pea, peb) and one mate-pair library (mp1). The parameter k specifies the k-mer length, and name is used to set the corresponding prefix of the output file name. The output of Abyss consists of outprefix-contigs.fa (which stores the contigs) and outprefix-scaffolds.fa, which stores the scaffolds generated by* de novo* assembly.

*(4) Velvet*. Velvet takes in short-read sequences, removes errors then produces unique contigs. The assembly process of Velvet consists of two stages: first, velveth takes in a number of read files, produces a hash index file, then outputs two files (Sequences and Roadmaps) in the output directory, which are necessary to the following program. Next, the core program, velvetg, builds and manipulates De Bruijn graphs for genomic sequence assembly.  Table 9 shows how to use the velveth commands. The hash_length (also known as k-mer length) corresponds to the length of the words being hashed. Here, the -fastq refers to the format of the read data. Supported read categories are short (default), shortPaired, short2, shortPaired2 (same as shortPaired, but for a separate insert-size library), long, and longPaired.  Table 10 shows how to use the velvetg commands. The parameters -cov_cutoff, -exp_cov, and -ins_length correspond to the coverage cutoff value, expected short-read k-mer coverage value, and expected insert length of the paired-end reads, respectively. If the scaffolding option is set to “yes” (default), Velvet tries to scaffold contigs. This command produces a fasta file which contains the sequences of contig and scaffold, and outputs some statistics.

*(5) Gap Filling*. Because the scaffolds are created by chaining multiple contig sequences, there may be gap regions between contigs in the scaffold sequences. In GAAP, GapCloser (https://sourceforge.net/projects/soapdenovo2/files/GapCloser) is used for filling gaps in the assembled scaffolds to increase the accuracy of assembly. GapCloser performs gap filling by realigning reads to the assembled scaffolds while considering relationships between paired-end reads.  Table 11 shows an example of using the GapCloser command. Option -a refers to the assembled scaffold file, and option -b refers to the config file. The config file has the same format as that used in the example in Table 4. Option -o refers to the final output file from running GapCloser, and option -l refers to the maximum read length. The final output file, genome.fasta, stores the new gap-filled scaffold sequences (Supplementary Figure S1).

#### 2.2.2. Transcriptome Assembly

Assembly software, based on the De Bruijn graph method, has been developed for assembling RNA reads such as Trinity (http://trinityrnaseq.github.io) and Oases (http://www.ebi.ac.uk/~zerbino/oases). GAAP uses Trinity, which generally shows relatively good performance. Trinity is composed of three modules: Inchworm, Chrysalis, and Butterfly. Inchworm, which is the assembly module, first generates a k-mer graph for RNA reads. Next, it generates the contigs by traversing the graph in a grid fashion. Chrysalis, which is a clustering module, constructs the clusters by combining contigs generated from alternative splicing or similar gene regions, and builds the De Bruijn graphs for each cluster. Finally, Butterfly optimizes the De Bruijn graphs and then traverses the optimized graphs to generate transcripts [31].

Table 12 shows an example of using the Trinity command. Here, the -* *-seqType option refers to the format of the read data, where fa stands for fasta, and fq stands for fastq. When running Trinity, it is essential to accurately indicate the direction of the strand for the RNA reads. The -* *-SS_lib_type option refers to the direction of the reads, where F is forward and R is reverse. The results file is trinity.fasta. This file stores the assembled transcript identifiers, lengths, paths in the De Bruijn graphs corresponding to the transcripts, and transcript sequences (Supplementary Figure S2).

### 2.3. Genome Annotation

Genome annotation refers to the process of identifying and attaching all the relevant features on a genome sequence. Genome annotation is divided into structural annotation and functional annotation. Structural annotation is the process of using assembled base sequence data to identify gene locations and structure, such as exons, introns, UTRs, and codons constituting these genes. Functional annotation refers to the process of identifying biochemical and metabolic activity, and cellular and physiological functions of gene products.

#### 2.3.1. Structural Annotation

*(1) Repeat Identification*. Repeats are found throughout genomic sequences and range from as small as 2 bp (simple repeats) to as large as 10 Kbp (interspersed repeats). Prior to gene prediction, it is important to mask repetitive elements, including low-complexity regions and transposable elements, because repeats will cause predictions of false homology. The repeats in input sequences should be masked by repeat-masking tools [32] such as RepeatMasker (http://repeatmasker.org) and RepeatRunner (http://www.yandell-lab.org/software/repeatrunner.html). Of these, RepeatMasker is widely used and employs a library of repeats drawn from Repbase (http://www.girinst.org/repbase/update/index.html) to recognize and mask repeat sequences [33].  Table 13 shows an example of using the RepeatMasker command. The -* *-species option indicates the name of the species in repeat library to be used for repeat masking. The input file, genome.fasta, contains scaffold sequences generated by the assembly and gap filling process.

*(2) Ab Initio Gene Structure Prediction*. After repeat masking, elements of gene structure, such as introns, exons, coding sequences (CDSs), start codons, and end codons, are predicted. To predict gene structure, Ab initio gene predictors use precalculated parameter values; these include distributions of intron-exon lengths and codon frequencies obtained from structural information on gene models such as* Caenorhabditis elegans*,* Drosophila melanogaster*, and humans. However, unless the newly sequenced genome is closely related to a gene model for which the precalculated parameters are available, the gene predictor needs to be trained on the genome that is under study. Augustus (http://augustus.gobics.de) and SNAP (http://korflab.ucdavis.edu/software.html) are representative examples of ab initio tools [34, 35].  Table 14 shows an example of using the Augustus command, which is available in GAAP. The input file is a repeat masked scaffold file, and the -* *-species option is used to set the name of a similar species whose genomic information can be used for structure prediction. In order to generate the results file in gff3 format, the -* *-gff3 option should be set to “on”.

*(3) Evidence-Driven Prediction of Gene Structure*. Most ab initio gene prediction tools can only find CDS structures, but cannot find UTRs or alternatively spliced transcripts. Thus, several recent methods are being used to predict gene structure based on external evidence. The evidence-driven method uses results, obtained by aligning expressed sequence tags (ESTs), protein sequences, and RNA-Seq data to a genome assembly, as external evidence. This method aims to improve the quality of gene prediction by integrating outputs of existing software tools (some of which are gene predictors) based on evidence alignments.

MAKER is an example of a representative evidence-driven structure prediction tool [36, 37]. MAKER (http://www.yandell-lab.org/software/maker.html) is a pipeline tool that combines several software tools in one; these tools include RepeatMasker, Exonerate, SNAP, Augustus, and Blast. Various parameters, which need to be set in MAKER configuration files, include maker_exe.ctl, maker_bopts.ctl, and maker_opt.ctl. Maker_exe.ctl sets the installation pathways for the programs used to run each program contained within MAKER (RepeatMasker, Exonerate, SNAP, Augustus, and Blast). Maker_bopts.ctl sets the thresholds for statistics filtering in the programs Blastn, Blastx, and Exonerate. Maker_opt.ctl contains information on the location of input files and on some of the parameters controlling decision making during the gene prediction. If trained gene predictors, such as SNAP and Augustus, are used in MAKER, they can be described in snaphmm and augustus_species options as shown in Table 15.

After setting all the relevant parameters to run MAKER, the maker command can simply be run as shown in Table 16.

**Figure 2:**
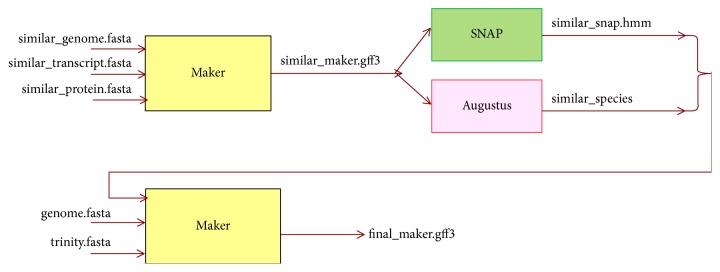
Workflow of running MAKER in GAAP. First, MAKER is run using scaffolds, transcripts, and proteins from similar species, and the results are used to train SNAP and Augustus. Next, the trained results are reinput into MAKER, along with assembled scaffolds and transcripts, to obtain the final annotation results.

However, in order to obtain even more accurate annotation results, the prediction results obtained from MAKER are used to train SNAP and Augustus, and results obtained through training are then reinput into MAKER; this process is repeated 3-4 times.  Figure 2 summarizes the method of running MAKER in GAAP.

The workflow is as follows:

  (Step 1) First, download the data (scaffolds, transcripts, proteins) from NCBI or wormbase for a species whose genome is similar to the genome to be annotated. Then, run the MAKER using only these data.
  (Step 2) The results from MAKER in previous step are used to train SNAP and Augustus. An hmm file from SNAP, and a species file corresponding to the new genome from Augustus, are generated as a result of this training.
  (Step 3) The hmm file and species file generated in Step (2) are reinput into MAKER, along with final scaffolds and transcripts obtained from assembly and repeat sequence masking. Here, the hmm file and species file can be described in maker_opt.ctl, as shown in Table 15.
  (Step 4) Steps (2)-(3) are repeated several times to obtain more accurate annotation results.

The results file from MAKER is in the gff3 format, and stores information about the number of genes predicted in the corresponding scaffolds, the locations of these genes, and the introns, exons, and CDSs (Supplementary Figure S3). The results obtained via MAKER can be inspected visually using GBrowse (https://sourceforge.net/projects/gmod/files), which shows gene structure predicted by each of the individual programs (SNAP, Augustus, Exonerate, Blast), as well as the final gene structure predicted by MAKER (Supplementary Figure S4).

*(4) Evidence-Based Consensus Gene Prediction*. When various gene prediction methods and tools are used to derive gene structure from a genome, it is essential to combine these results and obtain the single consensus gene structure. Consensus gene prediction tools include EvidenceModeler (EVM) (https://evidencemodeler.github.io), GLEAN (https://sourceforge.net/projects/glean-gene), and Evigan (http://www.seas.upenn.edu/~strctlrn/evigan/evigan.html). These tools extract a consensus gene structure by estimating the types and frequencies of errors generated by each source of gene evidence, and then choosing a combination of evidence that minimizes such errors [38, 39].

GAAP employs EVM, which is widely used. EVM combines various gene evidences, such as gene predictions and protein/transcript alignments, into weighted consensus gene structures. EVM allows the user to weight each evidence using a weights file. As shown in Table 17, the weights file consists of three columns: evidence class, type, and weight. The evidence class can be one of the following: ABINITIO_PREDICTION, PROTEIN, or TRANSCRIPT. ABINITIO_PREDICTION can use ab initio gene structure prediction tools, such as Augustus, Twinscan, SNAP, or GlimmerHMM. To improve the accuracy of annotation, EVM can use the results, obtained by aligning EST, full-length cDNA, or protein to the assembled genome, to extract consensus genes. In this case, PROTEIN can use a tool, such as GeneWise, to align a protein sequence to a genome for protein homology detection. TRANSCRIPT can use a tool, such as PASA, to align a full-length cDNA sequence to a genome. Weight refers to the weight value to be applied to each type of evidence. After preparing the weights file, EVM is run.

The process of running EVM consists of four phases: partitioning, execution, combining, and conversion. In the partitioning phase shown in Table 18, the input file is partitioned into smaller units, depending on factors such as memory capacity. Here, -* *-genome is used to set the assembled genome file, -* *-gene_predictions are used to set the predicted gene structure file, -* *-protein_alignments are used to set the protein file, and -* *-transcript_alignments are used to set the transcript file. A summary of the partitions is provided in the partitions_list.out file (parameter to -* *-partition_listing). In the execution phase shown in Table 19, a command list is generated first to enable executing EVM commands in parallel, in a grid computing environment, to improve performance. Next, the commands in the command list are executed. Here, -* *-weights sets the weights file, and -* *-output_file_name sets the output file for EVM results. In the combined phase shown in Table 20, the results for each small partitioned dataset, obtained in the execution phase, are joined into a single final result. Finally, in the conversion phase shown in Table 21, the final result file from the combining phase is converted into a standard gff3 format. After running the conversion phase, an evm.out.gff3 file is generated (Supplementary Figure S5). The conversion phase is optional.

*(5) Postprocessing to Add UTR Annotation*. The Program to Assemble Spliced Alignments (PASA) can update any gene structure annotations by correcting exon boundaries, and adding UTRs and alternatively spliced models based on assembled transcriptomic data. GAAP uses PASA (http://pasapipeline.github.io) to update the EVM consensus gene predictions, adding UTRs and modeling alternatively spliced isoforms.

PASA is run as follows. First, before running PASA, the gff3 result file, obtained from EVM (evm.out.gff3), is stored within a relational database (MySQL) (Table 22). In the database generation command, option -c is used to set the config file, option -g to set the assembled genome file, and option -P to set the final annotated results file (EVM results file). As shown in Table 23, the name of the database is set by the MYSQLDB option in the PASA config.file. In the example, the name of the generated database is myPasaDB.

The PASA execution command is shown in Table 24. Option -c refers to the config file, -g to the assembled genome file, and -t to the assembled transcriptome file. In this study, we used trinity.fasta, which stores the transcripts assembled using Trinity. The results of PASA contain UTR information and protein sequence information in #PROT lines (Supplementary Figure S6).

#### 2.3.2. Functional Annotation

Functional annotation is the process of attaching biological information to gene or protein sequences. Functional annotation can be divided into Blast-based homology search and gene ontology-based GO term mapping.

*(1) Homology Search*. To investigate gene function or predict evolutionary associations between related sequences, newly assembled sequences are compared with gene sequences with known functions to find sequences with high homology. Tools for homology search include Blast (https://blast.ncbi.nlm.nih.gov), TopHat (https://ccb.jhu.edu/software/tophat), and GSNAP (http://research-pub.gene.com/gmap). Blast2GO (https://www.blast2go.com) [40] is a pipeline tool that provides local and cloud-based methods of running Blast; these are named LocalBlast and CloudBlast, respectively. Because Blast performance is affected by the number and length of query sequences, it is preferable to use CloudBlast when performing a mass sequence alignment.

In GAAP, protein sequences obtained from the final results of PASA are used as query sequences, and Blastp is performed using the CloudBlast method provided in Blast2GO. In the result of Blastp, the meaning of each field is as follows: SeqName is the name of the query sequence; Description is a description of the mapped sequences; Length is the length of the query sequence; #Hits is the number of sequences mapped to the query sequence; e-value is the significance of the highest ranked mapped sequence; sim mean is the mean similarity of the mapped sequences; #GO is the number of mapped terms in GO ontology; GO list is the list of mapped terms in GO ontology; Enzyme list is the list of enzymes searched using GO terms; and InterPro Scan shows annotation data for the query sequence searched in a protein database (Supplementary Figure S7). In addition, Blast2GO provides various statistics about Blast results. For example, analyzing distribution statistics of top-hit species enables the user to find species most similar to the assembled genome.

*(2) GO Term Mapping*. Mapping is the process of retrieving GO terms associated with Hits (mapped sequences) obtained via Blast search. Gene ontology stores information about gene-related terms and relations between genes. Gene ontology is classified into three categories: biological process ontology, molecular function ontology, and cellular component ontology. GO database is a relational database to store and manage annotations describing information such as gene ontology, gene products, as well as functions and activity sites of gene products. GO database can be downloaded from AmiGO (http://amigo.geneontology.org/amigo).

In GAAP, we retrieve the GO terms by running the mapping tool provided in Blast2GO. By clicking the ID of a mapped GO term, it is possible to obtain detailed information about a GO term via linkage to the AmiGO site (Supplementary Figure S8). Blast2GO also provides the statistics of GO term frequency. Based on frequency distribution of mapped GO terms, we analyze the enrichment of GO categories (Supplementary Figure S9).

The analysis tool, provided in Blast2GO, can be used to analyze enzyme codes involved in Kyoto Encyclopedia of Genes and Genomes (KEGG) pathways and predict interactions between gene products. The KEGG pathways are network diagrams that represent interactions between numerous molecules and show metabolic activities. The nodes displayed as squares in the pathway diagrams represent enzymes; colored nodes represent enzymes that were retrieved from the genome assembled using GAAP. Here, #Seqs is the number of retrieved sequences associated with metabolic actions, and #Enzs is the number of retrieved enzymes related to metabolic activities (Supplementary Figure S10).

## 3. Discussion and Conclusions

We have designed and proposed GAAP, an assembly and annotation pipeline for whole-genome analysis. GAAP is composed of three stages of sequencing, assembly, and annotation. In this report, we detailed the analysis process at each stage. We described how to build analysis tools and databases that meet the needs of researchers. We also provided practical advice on how to set command-line parameters and explore the input/output data formats. The user guide is available at the GAAP website (http://GAAP.hallym.ac.kr).

Factors such as read length, read depth, insert size, and data quality affect the quality of genome assemblies. For the sequencing stage, we described how to carefully prepare sequencing data and how raw-read data are refined by using error-correction tools such as SOAPec and Markduplicates. For the assembly stage, we explained how contig and scaffold sequences for new species are generated using* de novo* assembly tools such as SOAPdenovo2, ALLPATHS-LG, Abyss 2.0, and Velvet without reference sequences. We also described how reliable gap filling is performed by using the GapCloser tool. The annotation stage was further divided into structural annotation and functional annotation. In structural annotation, we showed how to mask repeat sequences with RepeatMasker, and how to locate genes in scaffolds and identify introns, exons, and UTR structures within these genes using gene structure prediction tools such as Augustus, MAKER, EVM, and PASA. For the functional annotation step, we presented methods for identifying gene functions by homology search and ontology mapping using Blast2GO.

As a case study, a whole genome of* T. canis* was generated and deposited into GenBank, and is available at: http://www.ncbi.nlm.nih.gov/nuccore/LYYD00000000. The results of* T. canis* genome analysis derived using GAAP can be summarized as follows [2]. In the assembly stage of* T. canis*, N50 of the DNA sequences was 108 Kbp, and 10,853 scaffolds were obtained with a total length of 341 Mbp. The N50 of the RNA sequences was 940 bp, and 81,629 transcripts were obtained with a total length of 53,047 Kbp. In the gene structure prediction stage, 20,178 genes and 22,358 protein sequences were identified. Of the 22,358 protein sequences, 4,992 were newly observed in* T. canis*. Using homology search, gene ontology, and KEGG pathway analysis, we found that* T. canis* genes were most similar to those of* Ascaris suum* [41], and 127 enzymatic pathways were analyzed. These results obtained for* T. canis* were used to show the intermediate results generated at each stage of GAAP.

To the best of our knowledge, this is the first single pipeline encompassing generation of NGS read data, refinement, assembly, and annotation. Each step of the process is described in detail in this report. In this study, we compared and summarized various public software and biological databases required for each stage of the pipeline. For such software, we evaluated the commands, input/output data formats, and parameter settings. We also used a specific case study to show the intermediate results obtained at each stage. The pipeline described in this study enables a researcher without expertise in IT to perform a complete genome analysis. This is conducted by using each stage of the pipeline, examining intermediate results, and exploring the analyzed information for the generation of new ideas and hypotheses.

## Figures and Tables

**Table 1:** SOAPec commands.

KmerFreq_AR -k 17 -t 10 -p prefix readlist.txt
Corrector_AR -k 17 -l 3 -r 50 -t 10 prefix.freq.cz prefix.freq.cz.len readlist.txt

**Table 2:** FastqToSam command.

java -jar picard.jar FastqToSam F1=forward_reads.fq F2=reverse_reads.fq
O=unaligned_read_pairs.sam SM=sample001

**Table 3:** Markduplicates command.

java -jar picard.jar Markduplicates I=unaligned_read_pairs.sam O=output_duplicate.sam
M=output_duplicate_report.txt REMOVE_DUPLICATES=true

**Table 4:** SOAPdenovo configuration file.

[LIB]
max_rd_len=101
avg_ins=170
reverse_seq=0
asm_flags=3
rank=1
q1=DNAread170_1.fastq
q2=DNAread170_2.fastq
[LIB]
max_rd_len=101
avg_ins=2900
reverse_seq=1
asm_flags=2
rank=2
q1=DNAread2900_1.fastq
q2=DNAread2900_2.fastq

**Table 5:** SOAPdenovo command.

Soapdenovo all -s config.file -K kmerlength -o outprefix

**Table 6:** Preparing ALLPATHS input files.

PrepareAllPathsInputs.pl DATA_DIR=/data PLOIDY=1
IN_GROUPS_CSV=in_groups.csv IN_LIBS_CSV=in_libs.csv

**Table 7:** Running ALLPATHS command.

RunAllPathsLG PRE=<pre> REFERENCE_NAME=test.genome
DATA_SUBDIR=data RUN=run SUBDIR=test

**Table 8:** Abyss command.

abyss-pe k=kmerlength name=outprefix lib='pea peb' mp='mp1'
pea='DNAread170_1.fastq DNAread170_2.fastq'
peb='DNAread400_1.fastq DNAread400_2.fastq'
mp1='DNAread2900_1.fastq DNAread2900_2.fastq'

**Table 9:** Velveth command.

velveth output_directory/ hash_length -fastq
-shortPaired DNAread170_1.fastq DNAread170_2.fastq
-shortPaired2 DNAread400_1.fastq DNAread400_2.fastq
-longPaired DNAread2900_1.fastq DNAread2900_2.fastq

**Table 10:** Velvetg command.

velvetg output_directory/ -cov_cutoff auto -exp_cov auto -ins_length 170 -ins_length2 400
-ins_length_long 2900 -scaffolding yes

**Table 11:** GapCloser command.

GapCloser -a scaffold.scafSeq -b config.file -o genome.fasta -l readlength

**Table 12:** Trinity command.

Trinity -* *-seqType fq -* *-max_memory 50G -* *-left RNAread_1.fq -* *-right RNAread_2.fq
-* *-SS_lib_type FR

**Table 13:** RepeatMasker command.

RepeatMasker -* *-species species.name genome.fasta

**Table 14:** Augustus command.

augustus -* *-species=species.name -* *-gff3=on genome.fasta > output.file

**Table 15:** An example of maker_opt.ctl in MAKER.

#-* *-* *-* *-* *-Genome
genome=genome.fasta
#-* *-* *-* *-* *-EST Evidence
est=trinity.fasta
#-* *-* *-* *-* *-Protein Homology Evidence
protein=#protein sequence file in fasta format
#-* *-* *-* *-* *-Gene Prediction
snaphmm=similar_snap.hmm
augustus_species=similar_species

**Table 16:** MAKER command.

maker

**Table 17:** EVM weights file.

ABINITIO_PREDICTION augustus 1
ABINITIO_PREDICTION maker 1
PROTEIN genewise_protein_alignments 5
TRANSCRIPT PASA_transcript_assemblies 10

**Table 18:** EVM partitioning command.

EvmUtils/partition_EVM_inputs.pl -* *-genome genome.fasta
-* *-gene_predictions gene_predictions.gff3 -* *-protein_alignments protein_alignment.gff3
-* *-transcript_alignments transcript_alignment.gff3
-* *-segmentSize 100000 -* *-overlapSize 10000 -* *-partition_listing partitions_list.out

**Table 19:** EVM execution command.

EvmUtils/write_EVM_commands.pl -* *-genome genome.fasta -* *-weights weights.file
-* *-gene_predictions gene_predictions.gff3 -* *-protein_alignments protein_alignment.gff3
-* *-transcript_alignments transcript_alignment.gff3
-* *-output_file_name evm.out -* *-partitions partitions_list.out > commands.list

EvmUtils/execute_EVM_commands.pl commands.list

**Table 20:** EVM combining command.

EvmUtils/recombine_EVM_partial_outputs.pl -* *-partitions partitions_list.out -* *-output_file_name evm.out

**Table 21:** EVM conversion command.

EvmUtils/convert_EVM_outputs_to_GFF3.pl -* *-partitions partitions_list.out
-* *-output evm.out -* *-genome genome.fasta

**Table 22:** PASA database generation command.

scripts/Load_Current_Gene_Annotations.dbi -c config.file -g genome.fasta -P evm.out.gff3

**Table 23:** PASA configuration file.

# MySQL settings
MYSQLDB=myPasaDB

**Table 24:** PASA execution command.

scripts/Launch_PASA_pipeline.pl -c config.file -A -g genome.fasta -t trinity.fasta

## Data Availability

The data used to support the findings of this study are available from the corresponding author upon request.

## Supplementary Materials

Supplementary Materials Supplementary Table S1: comparison of all next-generation sequencing (NGS) Instruments (https://genohub.com/ngs-instrument-guide). Supplementary Figure S1: an example of a scaffold file generated after running GapCloser. The scaffold identifier and sequences in the scaffold are stored. Supplementary Figure S2: a transcript file generated after running Trinity. The assembled transcript identifier, length, pathways in the De Bruijn graph corresponding to the transcript, and transcript sequence are stored. Supplementary Figure S3: example results file from MAKER. The output file from MAKER is in gff3 format and stores information about the predicted gene locations within the relevant scaffold, as well as introns, exons, and coding sequences (CDSs) constituting the genes. Supplementary Figure S4: visualization of MAKER results using GBrowse. The black areas show predicted gene structures from programs used in MAKER such as SNAP, Augustus, Exonerate, and Blast. The blue area shows gene structures obtained in MAKER via integration of individual prediction results. Supplementary Figure S5: EVM results. Gene structures predicted using EVM store information on gene locations, as well as exons and coding sequences (CDSs) constituting the genes. Supplementary Figure S6: PASA results. The yellow portion shows information on new untranslated regions (UTRs) that have been added to gene-structure prediction results. In addition, protein sequence information can be examined in the #PROT line. Supplementary Figure S7: results screen from running Blastp in Blast2GO. SeqName is the name of the query sequence; Description is a description of the mapped sequences; Length is the length of the query sequence; #Hits is the number of sequences mapped to the query sequence; e-value is the e-value of the highest ranked mapped sequence; sim mean is the mean similarity of the mapped sequences; #GO is the number of mapped terms in gene ontology (GO); GO list is the list of mapped terms in GO; Enzyme list is the list of enzymes searched using GO terms; and InterPro Scan is annotation data for the query sequence searched in a protein database. Supplementary Figure S8: detailed information on gene ontology (GO) terms retrieved from the AmiGO site. Clicking the ID of a mapped GO term shows detailed information on the GO term via linkage with the AmiGO site. Clicking “show” provides a graph that shows the relationships between the GO term of interest and related GO terms. The square nodes in the graph represent the terms, and the edges indicated by arrows represent relationships between the nodes. Supplementary Figure S9: ontology-based functional annotation results. The distribution of gene ontology (GO) terms mapped based on biological process ontology, molecular function ontology, and cellular component ontology. Supplementary Figure S10: analysis of enzymes involved in purine metabolism. #Seqs is the number of sequences, retrieved from BLAST, which are associated with relevant metabolic activities; #Enzs is the number of retrieved enzymes related to the metabolic activity. The square nodes in the pathway diagram represent enzymes involved in the corresponding pathway. The colored nodes represent enzymes identified from the assembled genome.

## References

[1] Yandell M., Ence D. (2012). A beginner's guide to eukaryotic genome annotation. *Nature Reviews Genetics*.

[2] Kong J., Won J., Yoon J., Lee U., Kim J.-I., Huh S. (2016). Draft genome of toxocara canis, a pathogen resposible for visceral larva migrans. *The Korean Journal of Parasitology*.

[3] Cloonan N., Forrest A. R. R., Kolle G. (2008). Stem cell transcriptome profiling via massive-scale mRNA sequencing. *Nature Methods*.

[4] Miller J. R., Koren S., Sutton G. (2010). Assembly algorithms for next-generation sequencing data. *Genomics*.

[5] Metzker M. L. (2010). Sequencing technologies—the next generation. *Nature Reviews Genetics*.

[6] Mardis E. R. (2008). The impact of next-generation sequencing technology on genetics. *Trends in Genetics*.

[7] Scholz M. B., Lo C.-C., Chain P. S. G. (2012). Next generation sequencing and bioinformatic bottlenecks: The current state of metagenomic data analysis. *Current Opinion in Biotechnology*.

[8] Chaisson M., Pevzner P., Tang H. (2004). Fragment assembly with short reads. *Bioinformatics*.

[9] Pevzner P. A., Tang H., Waterman M. S. (2001). An Eulerian path approach to DNA fragment assembly. *Proceedings of the National Acadamy of Sciences of the United States of America*.

[10] Li R., Zhu H., Ruan J. (2010). *De novo* assembly of human genomes with massively parallel short read sequencing. *Genome Research*.

[11] Schröder J., Schröder H., Puglisi S. J., Sinha R., Schmidt B. (2009). SHREC: A short-read error correction method. *Bioinformatics*.

[12] Ilie L., Fazayeli F., Ilie S. (2011). HiTEC: accurate error correction in high-throughput sequencing data. *Bioinformatics*.

[13] Salmela L., Schröder J. (2011). Correcting errors in short reads by multiple alignments. *Bioinformatics*.

[14] Kao W.-C., Chan A. H., Song Y. S. (2011). ECHO: A reference-free short-read error correction algorithm. *Genome Research*.

[15] Warren R. L., Sutton G. G., Jones S. J. M., Holt R. A. (2007). Assembling millions of short DNA sequences using SSAKE. *Bioinformatics*.

[16] Dohm J. C., Lottaz C., Borodina T., Himmelbauer H. (2007). SHARCGS, a fast and highly accurate short-read assembly algorithm for de novo genomic sequencing. *Genome Research*.

[17] Jeck W. R., Reinhardt J. A., Baltrus D. A. (2007). Extending assembly of short DNA sequences to handle error. *Bioinformatics*.

[18] Margulies M., Egholm M., Altman W. E. (2005). Genome sequencing in microfabricated high-density picolitre reactors. *Nature*.

[19] Myers E. W., Sutton G. G., Delcher A. L. (2000). A whole-genome assembly of Drosophila. *Science*.

[20] Hernandez D., François P., Farinelli L., Østerås M., Schrenzel J. (2008). De novo bacterial genome sequencing: millions of very short reads assembled on a desktop computer. *Genome Research*.

[21] Luo R., Liu B., Xie Y. (2012). SOAPdenovo2: an empirically improved memory-efficient short-read de novo assembler. *GigaScience*.

[22] Butler J., MacCallum I., Kleber M. (2008). ALLPATHS: de novo assembly of whole-genome shotgun microreads. *Genome Research*.

[23] Gnerre S., MacCallum I., Przybylski D. (2011). High-quality draft assemblies of mammalian genomes from massively parallel sequence data. *Proceedings of the National Acadamy of Sciences of the United States of America*.

[24] Birol I., Jackman S. D., Nielsen C. B. (2009). De novo transcriptome assembly with ABySS. *Bioinformatics*.

[25] Jackman S. D., Vandervalk B. P., Mohamadi H. (2017). ABySS 2.0: resource-efficient assembly of large genomes using a Bloom filter. *Genome Research*.

[26] Zerbino D. R., Birney E. (2008). Velvet: algorithms for de novo short read assembly using de Bruijn graphs. *Genome Research*.

[27] Earl D., Bradnam K., John J. S. (2011). Assemblathon 1: a competitive assessment of de novo short read assembly methods. *Genome Research*.

[28] Bradnam K. R., Fass J. N., Alexandrov A. (2013). Assemblathon 2: evaluating de novo methods of genome assembly in three vertebrate species. *GigaScience*.

[29] Chu T., Lu C., Liu T., Lee G. C., Li W., Shih A. C. (2013). Assembler for de novo assembly of large genomes. *Proceedings of the National Acadamy of Sciences of the United States of America*.

[30] Khan A. R., Pervez M. T., Babar M. E., Naveed N., Shoaib M. (2018). A comprehensive study of de novo genome assemblers: current challenges and future prospective. *Evolutionary Bioinformatics*.

[31] Grabherr M. G., Haas B. J., Yassour M. (2011). Full-length transcriptome assembly from RNA-Seq data without a reference genome. *Nature Biotechnology*.

[32] Smith C. D., Edgar R. C., Yandell M. D. (2007). Improved repeat identification and masking in Dipterans. *Gene*.

[33] Jurka J. (2000). Repbase Update: A database and an electronic journal of repetitive elements. *Trends in Genetics*.

[34] Stanke M., Tzvetkova A., Morgenstern B. (2006). AUGUSTUS at EGASP: using EST, protein and genomic alignments for improved gene prediction in the human genome. *Genome Biology*.

[35] Korf I. (2004). Gene finding in novel genomes. *BMC Bioinformatics*.

[36] Cantarel B. L., Korf I., Robb S. M. C. (2008). MAKER: an easy-to-use annotation pipeline designed for emerging model organism genomes. *Genome Research*.

[37] Holt C., Yandell M. (2011). MAKER2: an annotation pipeline and genome database management tool for second-generation genome projects. *BMC Bioinformatics*.

[38] Haas B. J., Salzberg S. L., Zhu W. (2008). Automated eukaryotic gene structure annotation using EVidenceModeler and the Program to Assemble Spliced Alignments. *Genome Biology*.

[39] Liu Q., Mackey A. J., Roos D. S., Pereira F. C. N. (2008). Evigan: A hidden variable model for integrating gene evidence for eukaryotic gene prediction. *Bioinformatics*.

[40] Conesa A., Götz S., García-Gómez J. M., Terol J., Talón M., Robles M. (2005). Blast2GO: a universal tool for annotation, visualization and analysis in functional genomics research. *Bioinformatics*.

[41] Jex A. R., Liu S., Li B. (2011). *Ascaris suum* draft genome. *Nature*.

