# Supplementary material for: GAAP: A Genome Assembly + Annotation Pipeline
Source: Biomed Res Int. 2019 Jun 26;2019:4767354. doi: 10.1155/2019/4767354 (PMC6617929; doi:10.1155/2019/4767354)
Supplement: Supplementary Materials — Supplementary Table S1: comparison of all next-generation sequencing (NGS) Instruments (https://genohub.com/ngs-instrument-guide). Supplementary Figure S1: an example of a scaffold file generated after running GapCloser. The scaffold identifier and sequences in the scaffold are stored. Supplementary Figure S2: a transcript file generated after running Trinity. The assembled transcript identifier, length, pathways in the De Bruijn graph corresponding to the transcript, and transcript sequence are stored. Supplementary Figure S3: example results file from MAKER. The output file from MAKER is in gff3 format and stores information about the predicted gene locations within the relevant scaffold, as well as introns, exons, and coding sequences (CDSs) constituting the genes. Supplementary Figure S4: visualization of MAKER results using GBrowse. The black areas show predicted gene structures from programs used in MAKER such as SNAP, Augustus, Exonerate, and Blast. The blue area shows gene structures obtained in MAKER via integration of individual prediction results. Supplementary Figure S5: EVM results. Gene structures predicted using EVM store information on gene locations, as well as exons and coding sequences (CDSs) constituting the genes. Supplementary Figure S6: PASA results. The yellow portion shows information on new untranslated regions (UTRs) that have been added to gene-structure prediction results. In addition, protein sequence information can be examined in the #PROT line. Supplementary Figure S7: results screen from running Blastp in Blast2GO. SeqName is the name of the query sequence; Description is a description of the mapped sequences; Length is the length of the query sequence; #Hits is the number of sequences mapped to the query sequence; e-value is the e-value of the highest ranked mapped sequence; sim mean is the mean similarity of the mapped sequences; #GO is the number of mapped terms in gene ontology (GO); GO list is the list of mapped terms in GO; En [file 4767354.f1.doc]

**Supplementary**

Table S1: Comparison of all next-generation sequencing (NGS) Instruments (https://genohub.com/ngs-instrument-guide).

| Platform | Instrument | Reads/unit | Read Length (bp) | Read Type | Error Type |
| --- | --- | --- | --- | --- | --- |
| Illumina | NovaSeq 6000 S4 | 10,000,000,000 | 300 | SR &PE | substitution |
| Illumina | NovaSeq 6000 S3 | 6,600,000,000 | 300 | SR &PE | substitution |
| Illumina | NovaSeq 5000/6000 S2 | 3,300,000,000 | 300 | SR &PE | substitution |
| Illumina | NovaSeq 5000/6000 S1 | 1,600,000,000 | 300 | SR &PE | substitution |
| Illumina | NextSeq 500 High-Output | 400,000,000 | 300 | SR &PE | substitution |
| Illumina | HiSeq X | 375,000,000 | 300 | PE | substitution |
| Illumina | HiSeq 3000/4000 | 312,500,000 | 300 | SR &PE | substitution |
| Illumina | NextSeq 500 Mid-Output | 130,000,000 | 300 | PE | substitution |
| Illumina | HiSeq High-Output v4 | 250,000,000 | 250 | SR &PE | substitution |
| Illumina | HiSeq High-Output v3 | 186,048,000 | 250 | SR &PE | substitution |
| Illumina | HiSeq Rapid run v4 | 150,000,000 | 500 | SR &PE | substitution |
| Illumina | HiSeq Rapid Run | 150,696,000 | 300 | SR &PE | substitution |
| Illumina | HiScanSQ | 93,024,000 | 200 | SR &PE | substitution |
| Illumina | GAIIx | 42,075,000 | 300 | SR &PE | substitution |
| Illumina | MiSeq v3 | 25,000,000 | 600 | SR &PE | substitution |
| Illumina | MiniSeq High-Output | 25,000,000 | 300 | SR &PE | substitution |
| Illumina | MiSeq v2 | 16,000,000 | 250 | SR &PE | substitution |
| Illumina | MiniSeq Mid-Output | 8,000,000 | 300 | SR &PE | substitution |
| Illumina | MiSeq v2 Micro | 4,000,000 | 300 | SR &PE | substitution |
| Illumina | MiSeq v2 Nano | 1,000,000 | 500 | SR &PE | substitution |
| Ion | Proton I | 60,000,000 | 200 | SR | indel |
| Ion | PGM 318 | 4,000,000 | 400 | SR | indel |
| Ion | PGM 316 | 2,000,000 | 400 | SR | indel |
| Ion | PGM 314 | 400,000 | 400 | SR | indel |
| PacBio | PacBio Sequel | 370,000 | 20,000 | SR | indel |
| PacBio | PacBio RS II (P6) | 55,000 | 15,000 | SR | indel |
| Roche 454 | GS FLX+ / FLX | 700,000 | 700 | SR | indel |
| Roche 454 | GS FLX+ / FLX | 350,000 | 700 | SR | indel |
| Roche 454 | GS FLX+ / FLX | 125,000 | 700 | SR | indel |
| Roche 454 | GS FLX+ / FLX | 50,000 | 700 | SR | indel |
| Roche 454 | GS FLX+ / FLX | 20,000 | 700 | SR | indel |
| Roche 454 | GS FLX+ / FLX | 70,000 | 400 | SR | indel |
| SOLiD | 5500xl W | 266,666,667 | 100 | SR &PE | A/T Bias |
| SOLiD | 5500 W | 266,666,667 | 100 | SR &PE | A/T Bias |
| SOLiD | 5500 | 81,500,000 | 100 | SR &PE | A/T Bias |
| SOLiD | 5500xl | 81,500,000 | 100 | SR &PE | A/T Bias |

**
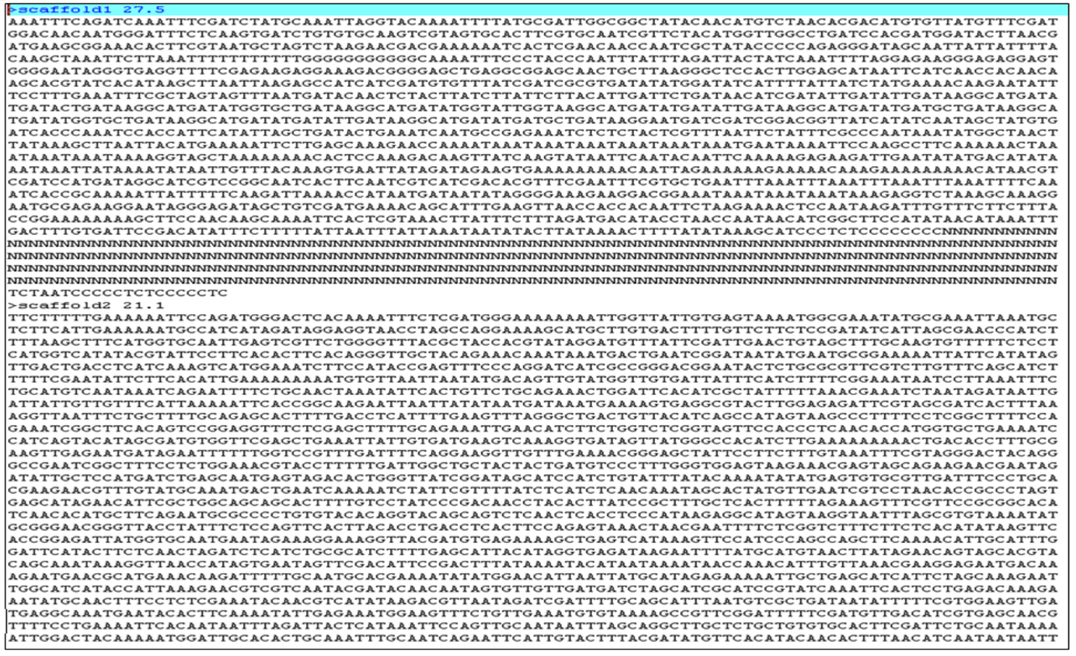
**

Figure S1: An example of a scaffold file generated after running GapCloser. The scaffold identifier and sequences in the scaffold are stored.

**
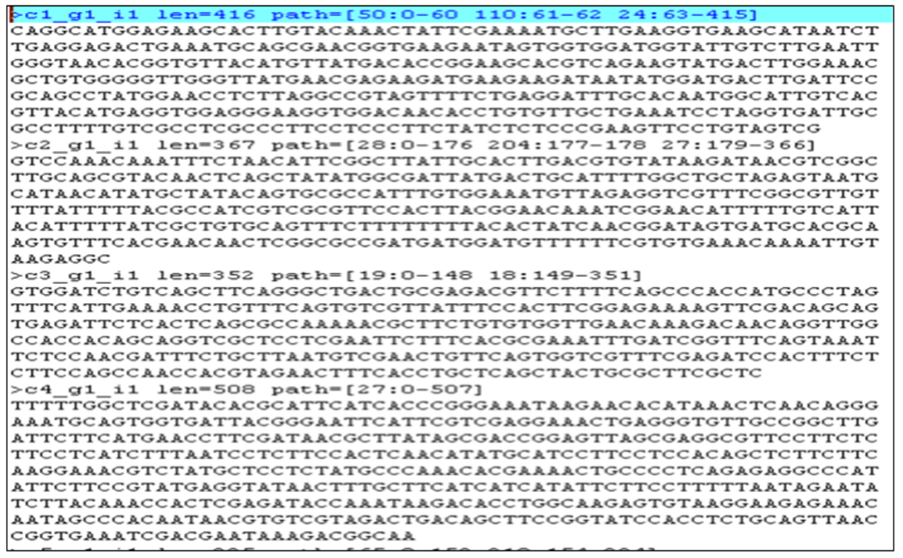
**

Figure S2: A transcript file generated after running Trinity. The assembled transcript identifier, length, pathways in the De Bruijn graph corresponding to the transcript, and transcript sequence are stored.

**
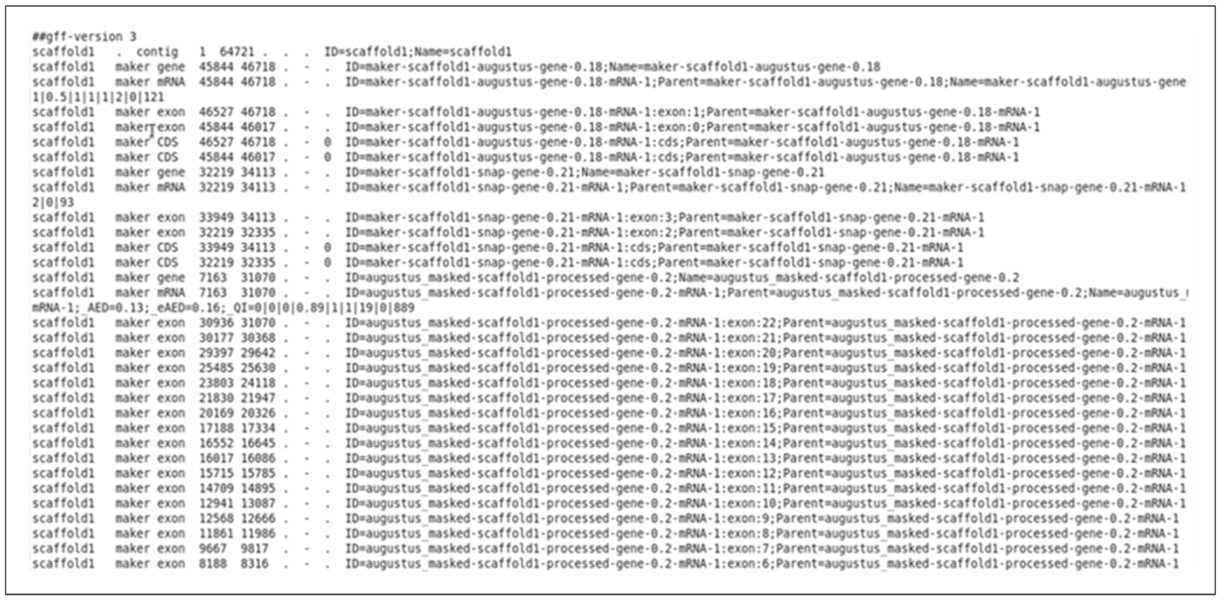
**

Figure S3: Example results file from MAKER. The output file from MAKER is in gff3 format, and stores information about the predicted gene locations within the relevant scaffold, as well as introns, exons, and coding sequences (CDSs) constituting the genes.

**
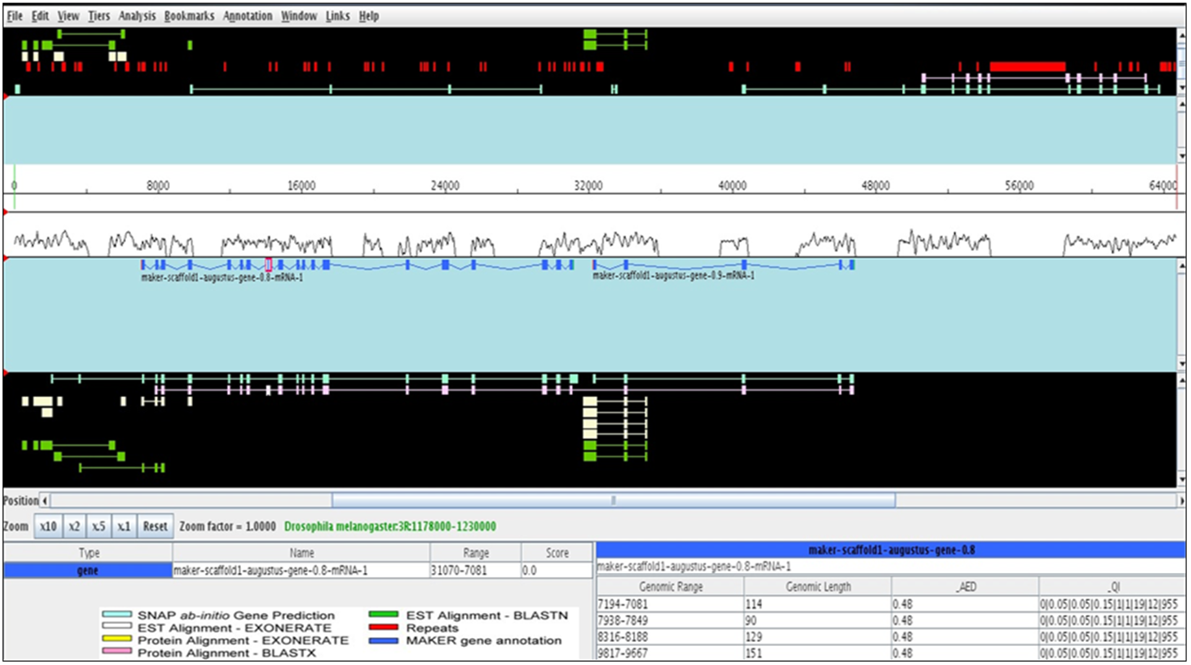
**

Figure S4: Visualization of MAKER results using GBrowse. The black areas show predicted gene structures from programs used in MAKER such as SNAP, Augustus, Exonerate, and Blast. The blue area shows gene structures obtained in MAKER via integration of individual prediction results.

**
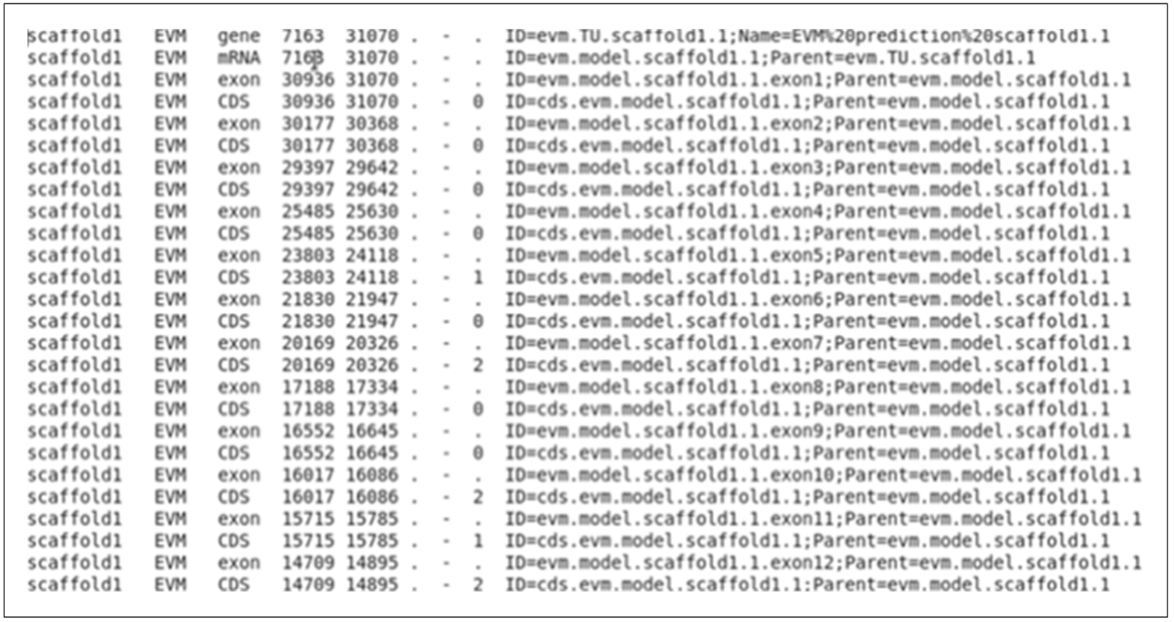
**

Figure S5: EVM results. Gene structures predicted using EVM store information on gene locations, as well as exons and coding sequences (CDSs) constituting the genes.

**
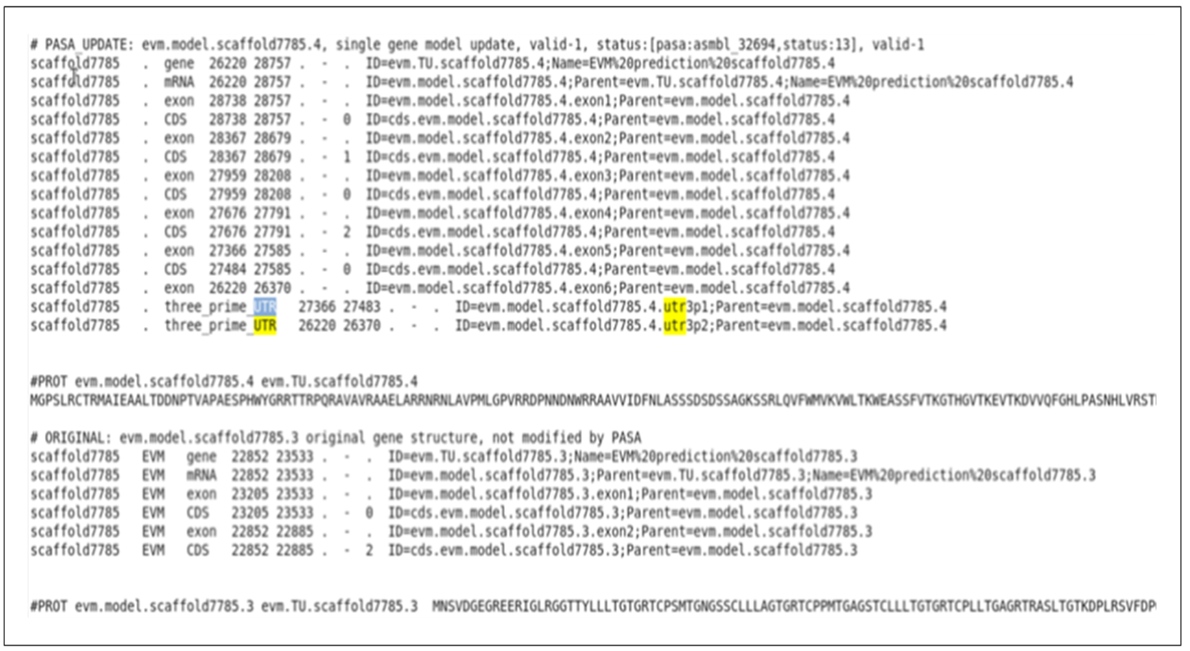
**

Figure S6: PASA results. The yellow portion shows information on new untranslated regions (UTRs) that have been added to gene-structure prediction results. In addition, protein sequence information can be examined in the #PROT line.

**
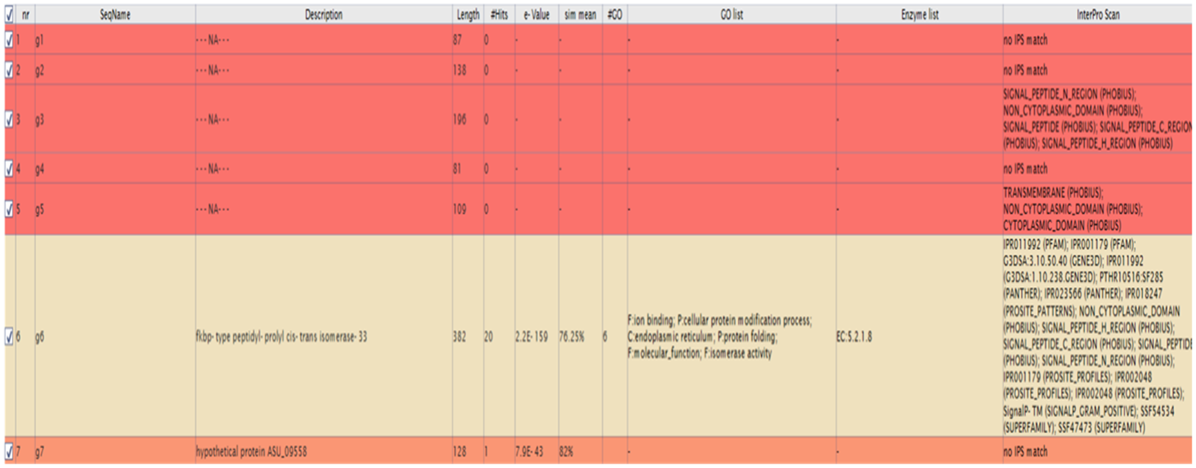
**

Figure S7: Results screen from running Blastp in Blast2GO. SeqName is the name of the query sequence; Description is a description of the mapped sequences; Length is the length of the query sequence; #Hits is the number of sequences mapped to the query sequence; e-value is the e-value of the highest ranked mapped sequence; sim mean is the mean similarity of the mapped sequences; #GO is the number of mapped terms in gene ontology (GO); GO list is the list of mapped terms in GO; Enzyme list is the list of enzymes searched using GO terms; and InterPro Scan is annotation data for the query sequence searched in a protein database.

**
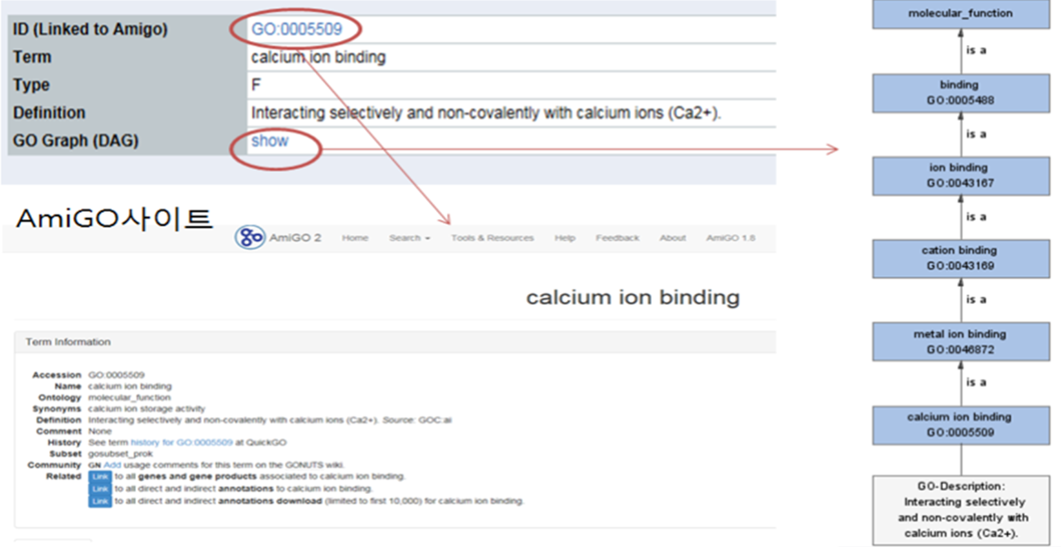
**

Figure S8: Detailed information on gene ontology (GO) terms retrieved from the AmiGO site. Clicking the ID of a mapped GO term shows detailed information on the GO term via linkage with the AmiGO site. Clicking “show” provides a graph that shows the relationships between the GO term of interest and related GO terms. The square nodes in the graph represent the terms, and the edges indicated by arrows represent relationships between the nodes.

**
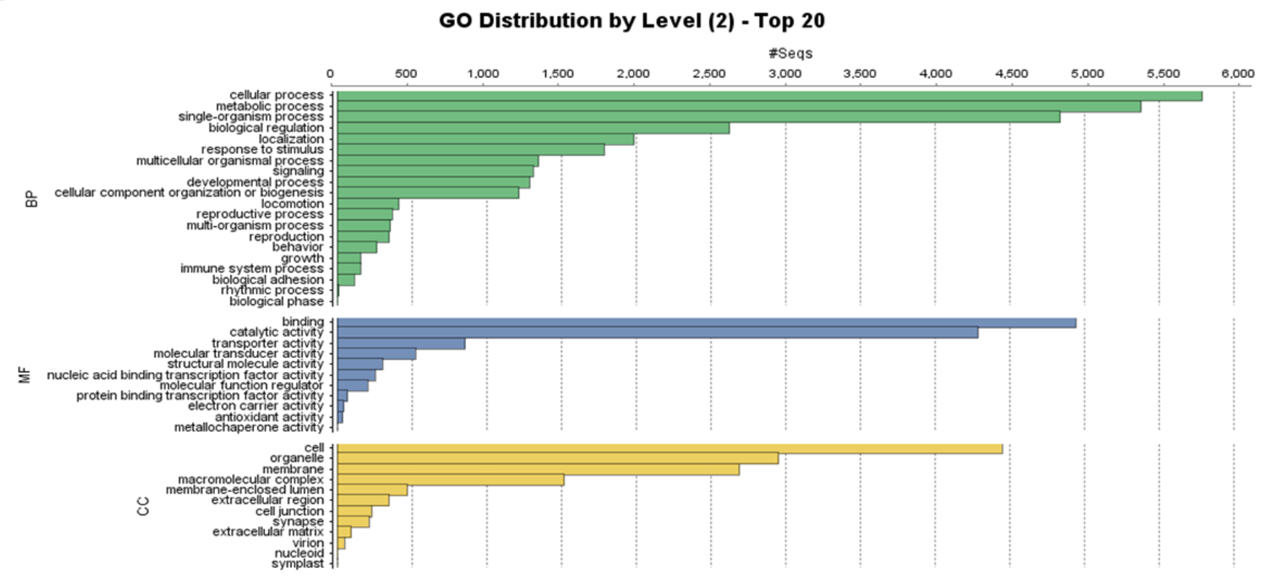
**

Figure S9: Ontology-based functional annotation results. The distribution of gene ontology (GO) terms mapped based on biological process ontology, molecular function ontology, and cellular component ontology.

**
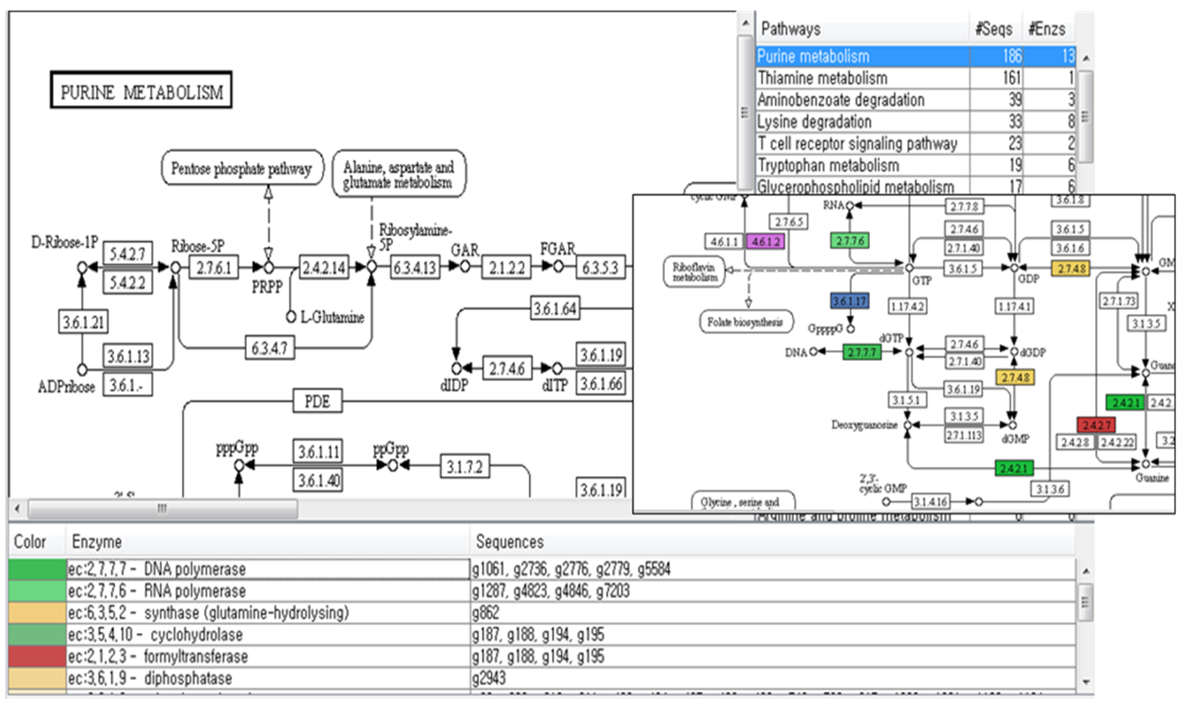
**

Figure S10: Analysis of enzymes involved in purine metabolism. #Seqs is the number of sequences, retrieved from BLAST, which are associated with relevant metabolic activities; #Enzs is the number of retrieved enzymes related to the metabolic activity. The square nodes in the pathway diagram represent enzymes involved in the corresponding pathway. The colored nodes represent enzymes identified from the assembled genome.
